# The comprehensive analysis of clinical trials registration for IgA nephropathy therapy on ClinicalTrials.gov

**DOI:** 10.1080/0886022X.2022.2048017

**Published:** 2022-03-10

**Authors:** Yan Cui, Ya-ling Zhai, Yuan-yuan Qi, Xin-ran Liu, Ya-fei Zhao, Fu Lv, Li-pei Han, Zhan-Zheng Zhao

**Affiliations:** a Nephrology Hospital, The First Affiliated Hospital of Zhengzhou University, Henan, China; b Institute of Nephrology, Zhengzhou University, Henan, China

**Keywords:** IgA nephropathy, treatment, clinical trials, ClinicalTrials.gov

## Abstract

**Objectives:**

IgA Nephropathy (IgAN) is common chronic kidney disease with a high incidence. This study aims to analyze comprehensively therapeutic clinical trials for IgAN registered on ClinicalTrials.gov.

**Methods:**

Therapeutic trials for IgAN registered on ClinicalTrials.gov. up to 15 August 2021 were obtained. The general characteristics, features of experimental design, treatment strategies, and some main inclusion criteria and outcome measures were accessed.

**Results:**

A total of 104 therapeutic clinical trials for IgAN were extracted on ClinicalTrials.gov up to 15 August 2021. Most of these trials explored the treatment for primary IgAN confirmed by renal biopsy in adults. Only 9% of all selected trials had results. Forty-five percent of trials recruited 50 or fewer participants, and 73% were adults or older adults. 99% of trials were interventional studies, and of all the interventional trials, 70% of trials were randomized, and 68% exercised a parallel assignment of intervention model. Immunosuppression was the most studied for the treatment of IgAN. Moreover, many novel agents had been increasingly studied in recent years. Furthermore, the inclusion criteria and primary outcome measures in these trials were diverse, and the level of proteinuria and change of proteinuria levels were the most used as inclusion criteria and primary outcome, respectively.

**Conclusions:**

The majority of therapeutic trials for IgAN were randomized, none masking and parallel-assignment interventional studies, primarily recruiting adult patients as research subjects. These trials had relatively small sample sizes and short observation. Thus, more large-scale, multicenter, and randomized controlled trials are still needed to improve the management for IgAN.

## 1. Introduction

Immunoglobulin A nephropathy (IgAN) is the most common primary glomerular disease in the world, with IgA depositing in the glomerular mesenterium as the main pathological feature [1]. IgAN may occur in patients of any age, but principally in young adults, and clinically characterized by asymptomatic hematuria with or without proteinuria. As a chronic kidney disease, IgAN almost cannot be cured completely presently and is one of the leading causes of end-stage renal disease (ESRD) [2]. In recent years, we have made great progress in the treatment of IgA nephropathy. The role of renin-angiotensin system (RAS) blockade in IgAN has been well-recognized, which can effectively control blood pressure and reduce urinary protein and thus slow the deterioration of renal function [3]. Therefore, an angiotensin-converting enzyme inhibitor (ACEi) or an angiotensin II-receptor blocker (ARB) is considered as a first-line treatment for IgAN. Additionally, more and more studies explore the role of immunosuppressive agents in the treatment of IgAN, such as corticosteroids, cyclophosphamide, and mycophenolate mofetil. But there still exists some controversy regarding their use in treating IgAN due to many side effects associated with immunosuppressive therapy [4]. Studies about exploring the effect of some novel drugs in IgAN have been the focus of researchers, such as Nefecon, complement inhibitors, drugs targeting cytokines and chemokines, and so on [5]. Nevertheless, 20–40% of patients with IgAN will still progress to ESRD, despite having received systematic management [6,7]. Therefore, improving the treatment for IgAN patients is still needed to establish an optimal management system to prolong renal survival and improve quality of life.

The importance of well-designed clinical trials, especially randomized controlled trials (RCTs), in establishing the first-rank treatment regimen for patients with IgAN cannot be overemphasized. ClinicalTrials.gov is one of the most important clinical trial registration centers in the world. It was developed by the US National Library of Medicine (NML) and US Food and Drug Administration (FDA) in 1997, with two main purposes: (1) Provide clinical trial information query services to patients, medical personnel, and the public; (2) Provide clinical trial registries services to medical researchers and institutions [8]. Therapeutic clinical trials of LN, T2DM, Melanoma, registered on ClinicalTrials.gov, have been thoroughly analyzed [9–11]. However, as the most dominant form of primary glomerulopathy, no comprehensive analysis studies of clinical trials for IgAN are found to date. Thus, this study aims to analyze comprehensively all therapeutic trials for IgAN registered on ClinicalTrials.gov, including general characteristics of trials, design characteristics of the study, therapeutic strategies, primary inclusion criteria, and outcome measures, and major research results, which will contribute to understanding systematically the current situation of clinical trials for IgAN and improving the therapeutic system.

## 2. Methods

### 2.1. Searching and filtrating clinical trials

All clinical trials for IgAN registered on ClinicalTrials.gov were extracted using the advanced search function with the only search criteria ‘IgA nephrology’ for ‘condition’ on 15 August 2021. Of all the searched trials, therapeutic trials for IgAN were singled out for comprehensive analysis. All following information was obtained: NCT number, title, study type, time of trials beginning, completion date of the study, observation period, number of participants, age, gender, study status, study phases, study designs, study results, Funded Bys, locations, primary outcome measures, eligibility criteria, with or not Data Monitoring Committee, U.S. FDA-regulated product and Individual Participant Data (IPD) sharing statement and so on.

### 2.2. Statistical analysis

The number and percentage of categorical data were computed. The categorical variables were presented as frequency or percentage. Descriptive analysis was used to represent the ordinary traits of all eligible clinical trials. All statistical analysis was implemented by using Word Processing System (WPS) office software.

## 3. Results

### 3.1. The general characteristics of eligible clinical trials

**Figure 1.**
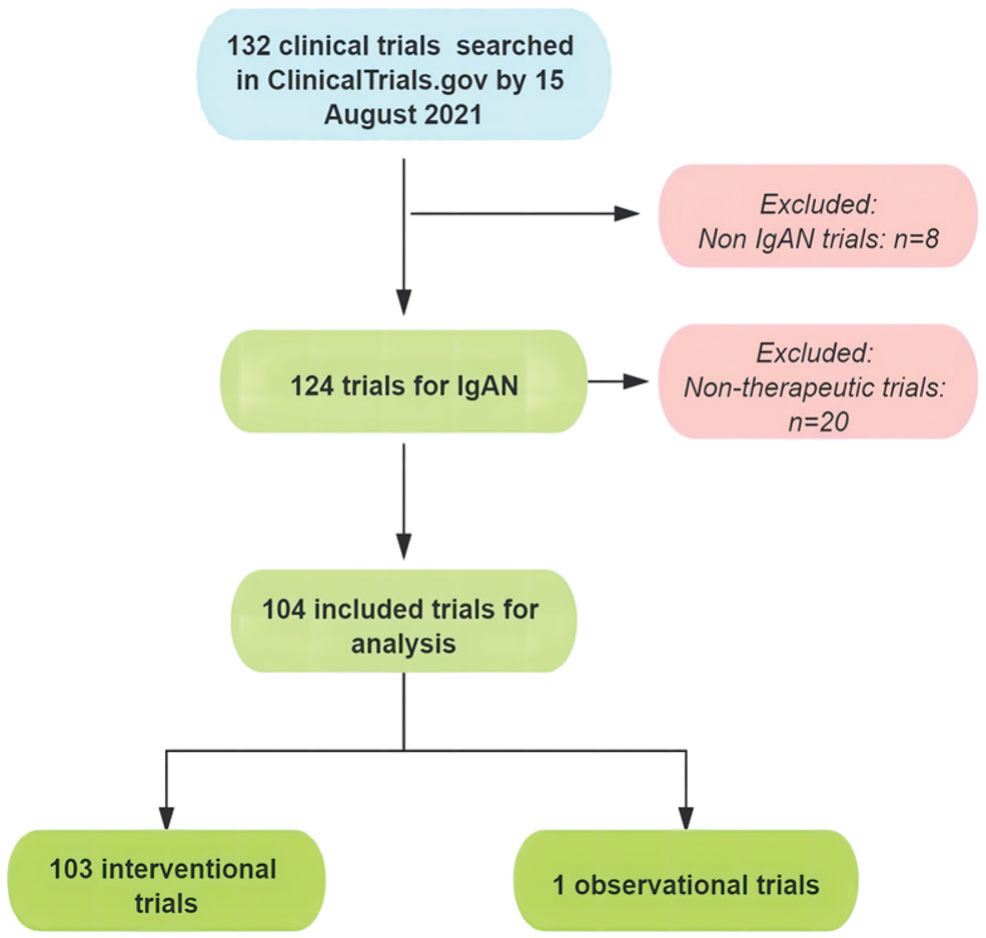
The flowchart of clinical trials extraction.

Up to 15 August 2021, we searched a total of 132 clinical trials. As shown in Figure 1, of all these trials, eight trials were not for IgAN, 20 trials were not therapeutic and thus were excluded. In the end, 104 trials were included and analyzed.

**Figure 2.**
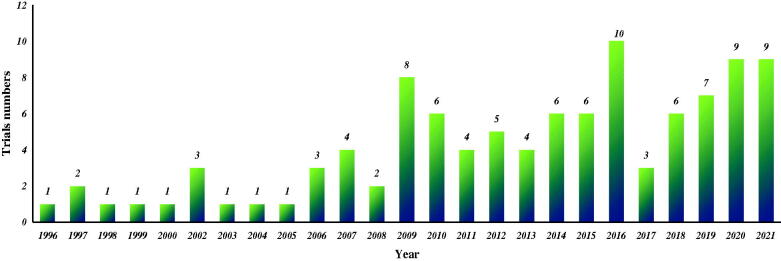
The number of clinical trials registered on ClinicalTrials.gov per year.

**Figure 3.**
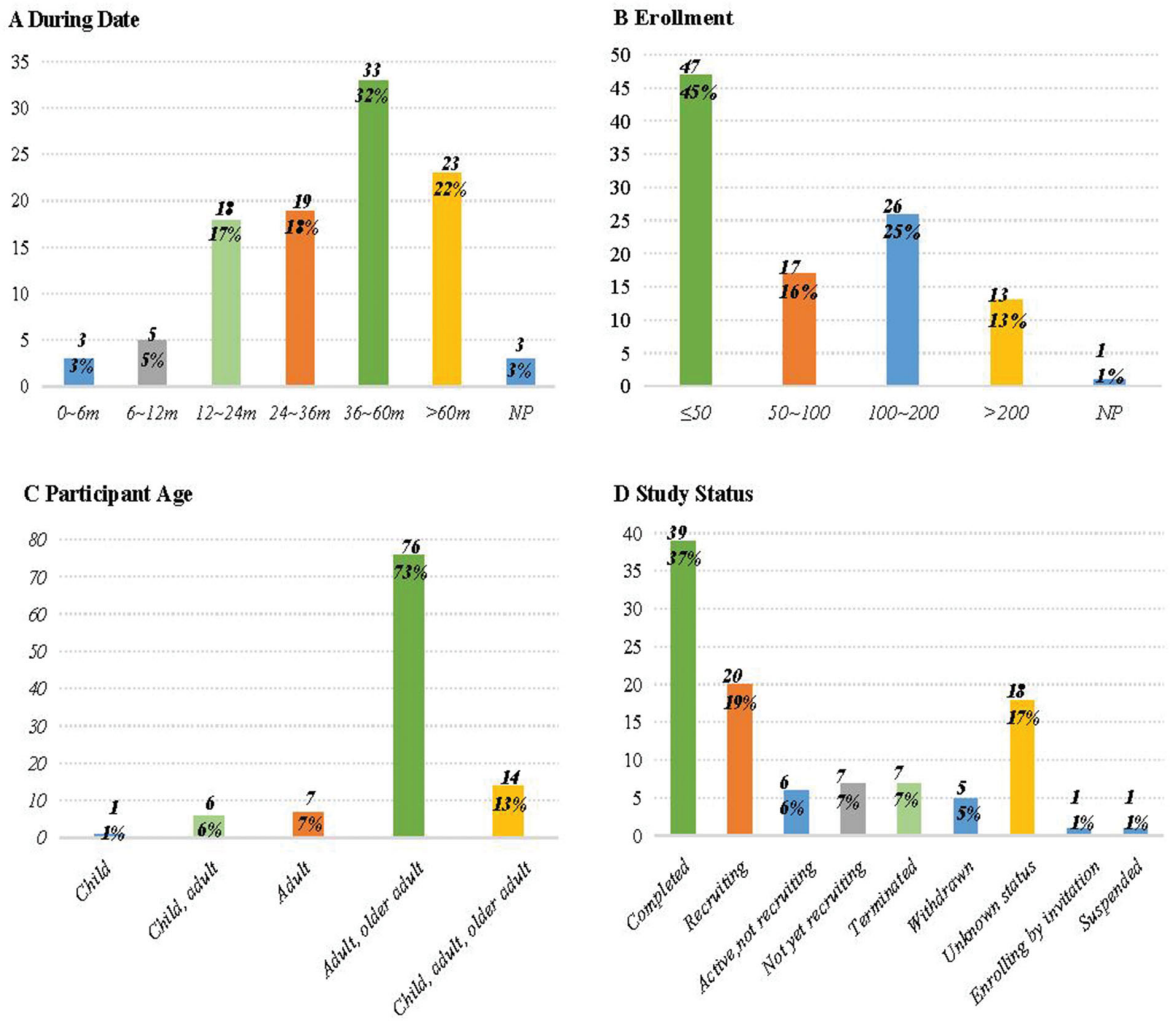
The general traits of selected clinical trials. (A) During date (observation period); (B) enrollment; (C) participant age; (D) study status. NP: not provided. *Notes*. Age (years old): child: <18; adult: ≥18 and <65; older adult: ≥65.

The general characteristics were shown in Figure 2, and we could find that the number of trials registered on ClinicalTrials.gov increased significantly after 2009 (*n* = 83), with the largest number in 2016 (*n* = 10). The general characteristics of included trials were presented in Figure 3. We found that of the 104 trials, 103 were interventional trials, while only one was observational. When the observation period was analyzed, about half of the trials were observed for more than 36 months (54%, *n* = 56), 18% for 24–36 months, 17% for 12–24 months, and 8% for <12 months. As for enrollment, 47 trials recruited fewer than 50 participants, 43 trials between 50 and 200, but only 13 trials more than 200. Moreover, of all these trials, 80% (*n* = 83) required only adults or older adults to be eligible for inclusion, 13% included both children, adults, and older adults but only 1% of trials required that only children be enrolled. In addition, as for the analysis of study status, it could be observed that only 37% (*n* = 39) trials were completed, and 19% were in recruiting, 6% were active not recruiting, 7% were not yet recruiting, 1% were enrolling by invitation. Additionally, 7% of trials were being terminated, 5% being withdrawn, 1% being suspended and 17% being in unknown status.

### 3.2. The characteristics of study design of selected clinical trials

**Figure 4.**
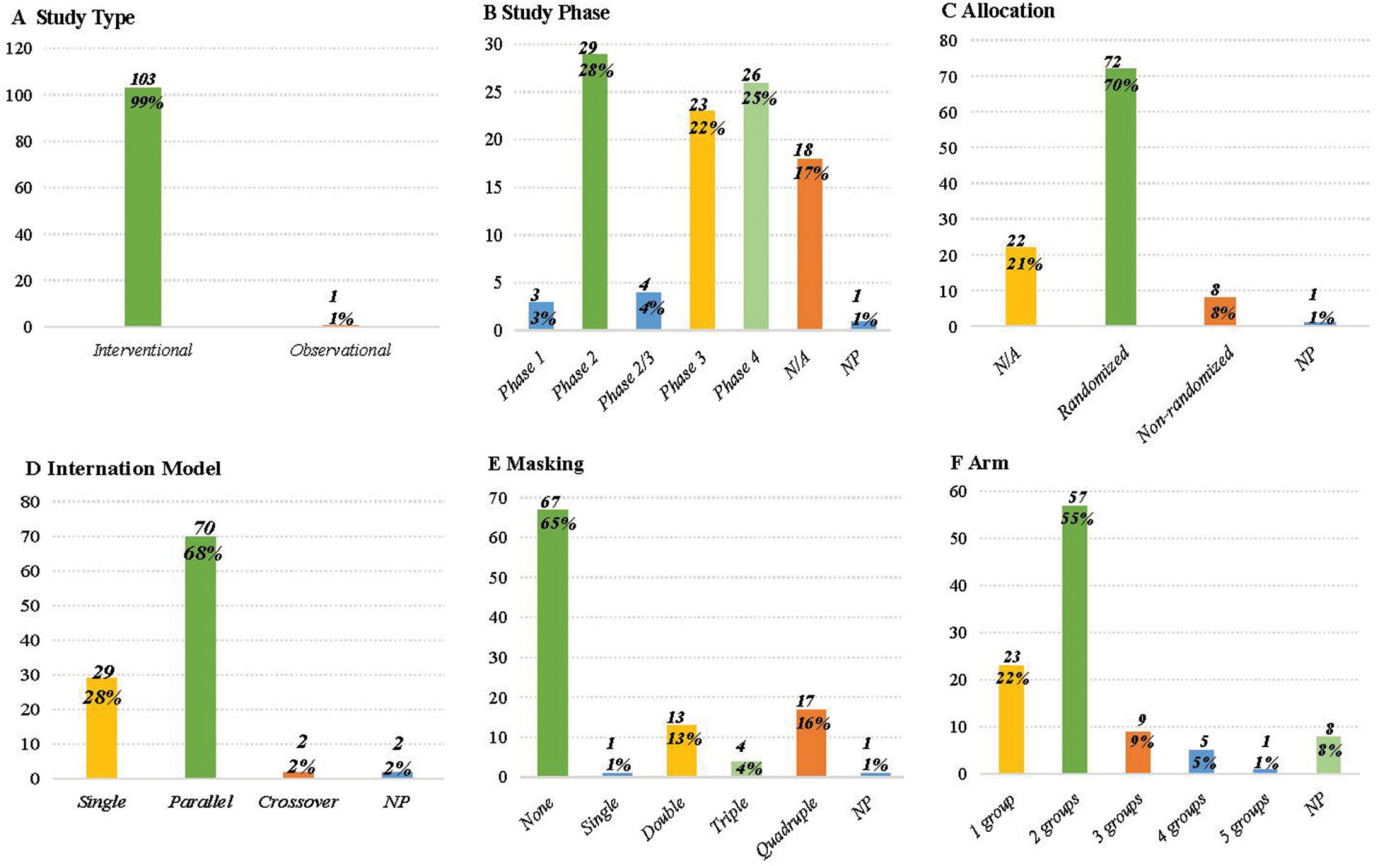
The characteristics of study design. (A) Study type; (B) study phase; (C) allocation; (D) intervention model; (E) masking; (F) arm. NP: not provided; N/A: not applicable.

The study design of 104 clinical trials targeting the treatment IgAN was anatomized and summarized, and shown in Figure 4. We found that about half of these clinical trials (54%, *n* = 56) were in phase 2 or phase 3, 25% in phase 4, and 17% were not applicable (N/A). Of 103 interventional studies, most were randomized allocation trials (*n* = 72, 70%), and 8% (*n* = 8) were non-randomized. In addition, 68% (*n* = 70) trials were a parallel assignment for the intervention model, 65% (*n* = 67) were not masked and 55% of trials were divided into two groups. When it came to the observation trial, it was a retrospective study on the role of tonsillectomy in treating IgAN.

### 3.3. Other more details about the clinical trial features of IgA nephropathy

**Figure 5.**
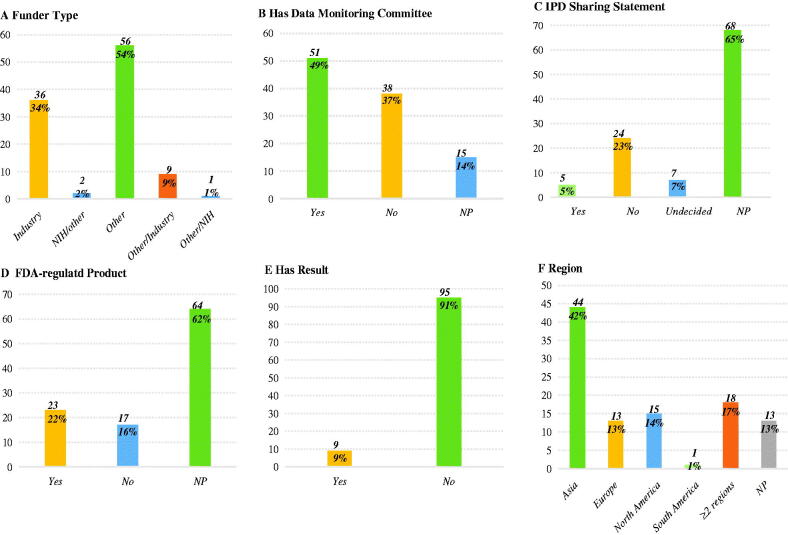
More elaborate features of selected clinical trials. (A) funder type; (B) data monitoring committee; (C) IPD sharing statement; (D) FDA-regulated product; (E) study results; (F) Region. NP: not provided.

We analyzed additional characteristics about included trials of IgAN, and the results of our analysis were presented in Figure 5. We discovered that these clinical trials received the most financial aid from other types of funds (*n* = 56, 54%), followed by the funds of industry (*n* = 36, 34%). Of all trials, 49% of trials (*n* = 51) had a data monitoring committee (DMC), except for 14% that did not provide information. In addition, only 5% of trials (*n* = 5) planned to share IPD and 22% of trials (*n* = 23) studied FDA-regulated products, while most trials did not provide the two information mentioned above (*n* = 68, 65% and *n* = 64, 62%, respectively). Furthermore, only 9% (*n* = 9) had study results. In terms of the regions that trials were conducted, we found that 70% (*n* = 73) trials were implemented on a single continent, and among these continents, Asia was the most primary single continent on which clinical trials were conducted (*n* = 44, 42%). Additionally, 17% of trials were implemented on two or more continents.

### 3.4. Analysis and summarization of therapeutic strategies for IgAN in included clinical trials

As shown in Table 1, We analyzed the treatment strategies of IgAN in these clinical trials. These strategies could be broadly grouped into the following categories: supportive treatment, immunosuppressive therapy, novel targeted-release corticosteroid, Chinese medicine, regulating gut microbes, integrative medicine, operation, targeting complement components, targeting cytokines, and chemokines, targeting endothelin, targeting signal path, and some other novel drugs. Among these, we found that immunosuppressive therapy was the most studied, followed by supportive management. Among the immunosuppressive agents, the most researched was corticosteroids (*n* = 9), followed by mycophenolate mofetil (MMF) (*n* = 6). In addition, some drugs combinations were explored for the treatment of IgAN, such as corticosteroids combined with cyclophosphamide (CTX) (*n* = 4), or with RAS blockade (*n* = 3), or with azathioprine (*n* = 2), or with MMF (*n* = 2), or with leflunomide (*n* = 1), or with CTX and Chinese medicine (*n* = 1) and so on. What’s more, a novel targeted-release corticosteroid, Nefecon, was also repeatedly studied for IgAN, recently (*n* = 4). In the supportive management, renin-angiotensin system (RAS) blockades were investigated the most (*n* = 15). It is precise because many studies have confirmed the protective effects of RAS blockade on patients with IgAN that angiotensin-converting enzyme inhibitor (ACEI) or an angiotensin II-receptor blocker (ARB) are used as the first-line treatment for IgAN in clinical practice. Furthermore, many other supportive managements were also investigated for the therapy of IgAN, such as hydroxychloroquine, statins, omega-3 fatty acids, diet intervention, calcitriol, and so on. Another relatively well-studied trials were drugs targeting complement components. Three trials explored LNP023, two trials explored OMS72, and IONIS-FB-LRx, Cemdisiran, CCX168, Ravulizumab, and APL-2 were studied in one trial, respectively. In addition, 11 trials studied drugs targeting cytokines and chemokines, five trials studied drugs targeting endothelin, and four trials investigated drugs that target the signal path. Some medicines studied in seven clinical trials were classified as other novel drugs.

**Table 1.** Treatment strategy for IgAN in these clinical trials.

Types	Name of drugs	Number
Supportive management		29
	RAS blockade	15
	Hydroxychloroquine	3
	Statin	1
	Omega-3 Fatty Acids	2
	Diet intervention	3
	Calcitriol	3
	Paricalcitol	1
	Weight reduction	1
Immunosuppression		31
	Steroids	9
	Steroids + RAS blockade	3
	CTX	1
	CTX + Steroids	4
	CTX + Steroids + Chinese Medicine	1
	Azathioprine + Steroids	2
	MMF	6
	MMF + Steroids	2
	Tacrolimus	1
	Sirolimus	1
	Leflunomide + Steroids	1
Novel targeting corticosteroid	Nefecon	4
Chinese medicine		4
	Abelmoschus manihot	1
	Reh-acteoside	1
	Tripterygium Wilfordii Hook.f	2
Regulating gut microbes		2
	Fecal Microbiota Transplantation (FMT)	1
	Probiotics	1
Integrative medicine	Shentong Granules + Prednisone	2
Operation		3
	Tonsillectomy	2
	Plasma Exchange	1
Targeting complement components		10
	LNP023	3
	IONIS-FB-LRx	1
	OMS721	2
	Cemdisiran	1
	CCX168	1
	Ravulizumab	1
	APL-2	1
Targeting cytokines and chemokines		11
	Telitacicept	2
	Atacicept	2
	Blisibimod	3
	BION-1301	2
	VIS649	1
	CCL2-LPM	1
Targeting endothelin		5
	Atrasentan	2
	Sparsentan	3
Targeting signal path		4
	AVB-S6-500	1
	Fostamatinib	2
	Bardoxolone Methyl	1
Other novel drugs		7
	Acthar^®^	3
	Veclcade	1
	SM101	1
	ATG-F	1
	ADR-001	1

### 3.5. Main inclusion criteria in these included clinical trials

In most clinical trials, strict inclusion criteria would be established to ensure that the trials could be carried out smoothly. Without exception, the inclusion criteria of these included trials in our study were rich and varied. Shown in Table 2, We mainly summarized several inclusion or exclusion criteria that occurred frequently in the included trials and were commonly used in clinical practice, including the diagnosis criteria of IgAN, the level of 24-h urine total protein quantification (24hUTP), urine protein creatinine ratio (uPCR), serum creatitine (Scr), and estimated glomerular rate filtration (eGFR). Among these clinical trials, most required patients with IgAN confirmed by renal biopsy (*n* = 75), and clinical evaluation combined with renal biopsy was required in eight trials. Sixty-six trials clarified criteria for the level of 24hUTP in enrolled patients. Among those, the level of ≥1 g/24 was included in 34 trials, ≥0.5 g/24 h in five trials, and ≥0.75 in four trials. The inclusion criteria of uPCR level were interpreted in 24 trials, and eight trials determined that the level of uPCR was ≥1 g/g, five trials determined the level ≥0.5 g/g, and three trials determined the level ≥0.75 g/g. In addition, only 19 trials defined the inclusion criteria of serum creatitine, and the level of ≤265.2 μmol/L was the most mentioned (*n* = 6). What’s more, eGFR levels of included patients were required in 71 trials. Almost half of our extracted trials required the inclusion of patients with the level of eGFR ≥ 30 mL/min/1.73 m^2^, followed by six trials requiring patients with eGFR level ≥45 mL/min/1.73 m^2^.

**Table 2.** Primary inclusion criteria of these clinical trials for IgAN.

Main inclusion criteria	Details	Number
Diagnosis of IgAN		
	Biopsy-proven IgAN	75
	Clinical evaluation and renal biopsy	8
	NP	21
Level of 24hUTP (g/24 h)		66
	≥2	1
	≥1	34
	>0.8	1
	≥0.75	4
	>0.7	1
	≥0.5	5
	>0.3	1
	1–7	1
	1–6	1
	1–3.5	3
	1–3	2
	1–2.5	1
	0.75–3.5	2
	0.5–3.5	1
	0.5–1.5	1
	0.5–3	1
	0.3–3.5	1
	0.15–3	1
	<3.5	1
	<0.5	2
	0.3–1/>1	1
Level of uPCR (g/g)		24
	≥2.5	1
	≥1	8
	≥0.8	1
	≥0.75	3
	≥0.5	5
	>0.3	1
	0.75–6	1
	0.3–3	1
	0.5–1	1
	0.3–1	1
	≤2.5	1
Serum creatinine (umol/L)		19
	<442	1
	<400	1
	≤309.4	1
	≤265.2	6
	<200	2
	≤176.8	1
	≤132.6	2
	<120	2
	≥176.8	1
	≥200	1
	≥106.08 (female)/≥123.76 (male)	1
eGFR (ml/min/1.73 m^2^)		71
	≥90	1
	≥80	1
	≥60	4
	≥50	2
	≥45	6
	≥40	3
	≥30	34
	≥20	3
	≥15	1
	≥50/45	1
	≥45/30–45/20–30	1
	45–90	1
	30–120	1
	30–90	2
	30–60	2
	30–45	1
	25–50	1
	20–120	1
	15–90	1
	15–60	2
	15–50	1
	<60	1

Abbreviations: 24hUTP: 24-h urine total protein quantification; uPCR: urine protein creatinine ratio; Scr: serum creatitine; eGFR: estimated glomerular rate filtration.

*Notes*. Inclusion criteria for the level of 24hUTP (g/24 h): ≥1 (*n* = 18) and >1 (*n* = 16); ≥0.75 (*n* = 1) and >0.75 (*n* = 3); ≥0.5 (*n* = 2) and >0.5 (*n* = 3); uPCR (g/g): ≥1 (*n* = 5) and >1 (*n* = 3); ≥0.75 (*n* = 1) and >0.75 (*n* = 2); ≥0.5 (*n* = 3) and >0.5 (*n* = 2); Scr (mmol/l): ≤265.2 (*n* = 1) and <265.2 (*n* = 5); eGFR (ml/min/1.73 m^2^): ≥60 (*n* = 2) and ≥60 (*n* = 2); ≥45 (*n* = 4) and >45 (*n* = 2); ≥40 (*n* = 1) and >40 (*n* = 2); ≥30 (*n* = 23) and >30 (*n* = 11); ≥20 (*n* = 2) and >20 (*n* = 1).

### 3.6. Primary outcome measures in the included clinical trials

The primary outcome measures of eligible trials in our study were comprehensively analyzed and outlined, and the results were presented in Table 3. We could find that change of proteinuria from baseline was the most regarded as an outcome in all included trials (*n* = 40). Among the 40 clinical trials, 24hUTP was used as an outcome in eight trials, uPCR was used in 19 trials and both were used in one trial. The remaining 12 trials used proteinuria as an outcome, without specifying particular indices. The outcome of 15 trials was the level of proteinuria, which also included the levels of 24hUTP, uPCR, or proteinuria without stating specific indices. As known, drug safety is a widely concerned practical problem in clinical practice. In our analysis, the observed outcomes of 13 trials were the safety of drug treatment studied and the onset of adverse events (AEs). In addition, remission rate after treatment was regarded as a primary outcome in 13 trials. Among those, complete remission (CR) and/or partial remission (PR) were included in nine trials, and five trials clarified the definition of CR and PR and they were different. The other four trials only used CR as a primary outcome, and only one trial defined CR. In addition, progression to ESRD was used as one of the primary outcome measures in 10 trials, the onset of death was used in six trials, change from baseline of eGFR was used in five trials and 50% rise from baseline of serum creatitine was used in five trials. Furthermore, one of the primary outcomes in four included trials was the emergence of renal replacement therapy, which covered dialysis and/or renal transplantation.

**Table 3.** The primary outcome measures in the clinical trials.

Outcome measures	Number
Change from baseline of proteinuria	40
Proteinuria level	15
Decline of proteinuria	4
Serum creatitine level	2
Doubling of serum creatinine	3
50% rise from baseline of Scr	5
Change from baseline of eGFR	5
eGFR level	6
Onset of adverse events	13
Blood pressure	3
CR	4
Remission rate (CR/ PR)	9
Recurrence	1
Onset of renal replacement treatment	4
Onset of ESRD	10
Onset of death	6

Scr: serum creatitine; eGFR: estimated glomerular rate filtration; CR: complete remission; PR: partial remission; ESRD: end-stage renal disease.

### 3.7. Analysis of the results of clinical trials in the selected trials

**Figure 6.**
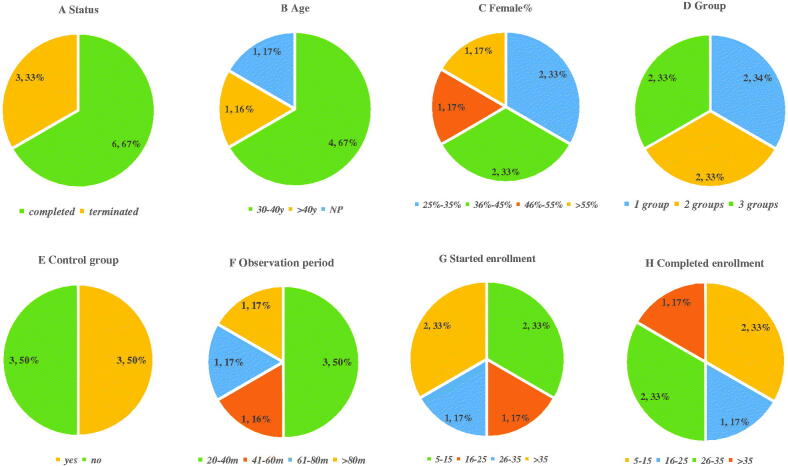
Characteristics of trials with results. (A) Study status; (B) mean or median age (years old); (C) Female%; (D) Group; (E) has control group; (F) observation period (months); (G) enrollment who starting trial; (H) enrollment who completing trial. NP: not provided.

To understand the research results of clinical trials on treating IgAN, as well as the characteristics and advantages, and disadvantages of the trials, we further analyzed the clinical trials with experimental results. As shown in Figure 6, nine trials had resulted in all included trials, which included six trials were completed and three were terminated. One trial was terminated for extremely slow enrollment, one was stopped for lack of effect and one trial provided no information about the reasons. Thus, six completed trials with results were analyzed detailedly. The mean or median age of patients in four trials ranged from 30 to 40 years old. Two trials were divided into one group, two into two groups, and two into three groups, but half of the trials (*n* = 3) did not have a control group. 50% (*n* = 3) trials were observed between 20 and 40 months. Only two trials had more than 35 enrollments when the study was started, and only one trial had over 35 enrollments who completed trials. In addition, four trials provided the reasons for not completing trials (data not shown). Furthermore, our analysis of the results showed that one of the studies was allopurinol, one was Bortezomib, one was Rituximab, one was Fostamatinib and two was ACTH Gel. Only the study about Fostamatinib provided the statistical analysis on clinicalTrials.gov, and the differences were statistically insignificant. In addition, we searched the full texts of four trials on PubMed.gov. Rituximab could not significantly improve patients’ renal function and proteinuria after 1 year of follow-up, and allopurinol could not improve renal function and proteinuria but played a role in controlling blood pressure [12,13]. Bortezomib reduced proteinuria in IgAN patients, but the study was non-randomized, had a small sample size, and lacked a control group [14]. Additionally, proteinuria remissions were also achieved in some patients with refractory nephropathy treated with ACTH Gel [15].

## 4. Discussion

In this study, we comprehensively analyzed and generalized the characteristics, therapeutic strategies studied, main inclusion criteria, and primary outcome measures and results of therapeutic clinical trials for IgAN registered on ClinicalTrials.gov. Through a careful analysis of all trials included in our study, we found a significant increase in the number of therapeutic trials registration for IgAN since 2009. This might be attributed to the International Committee of Medical Journal Editors (ICMJE) putting forward the statement in 2004 that all clinical trials should be registered at the time of recruiting volunteers or even earlier. Also, this requirement was regarded as one of the conditions for considering whether trials could be published by the ICMJE member journals [16].

IgAN is a lifelong chronic disease and has a 5-15% risk of progression to ESRD after 5 years since diagnosis [17]. The disease course of IgAN is various, ranging from persistent and asymptomatic mild cases to severe cases with rapid disease progression. Therefore, short, medium, and long periods were all covered in the observation period of these selected trials. Surprisingly, about 22% of trials were observed for over five years. In addition, 45% of trials had a small sample size of <50 enrollments. Results from large-scale, multicenter, and randomized trials are more convincing and reliable, and large-scale clinical trials contribute to meeting experiment outcomes compared with single-center clinical trials with a small sample size [18]. In our analysis of participants' age, the majority of trials explored the treatment of IgAN in adults (>18 years old), but very few studied that only in children. Although young adult males are the main group of IgAN patients, there are many differences in the disease management between children and adults [19]. Furthermore, considered drug tolerance, more caution is demanded in the treatment of IgAN in children. Thus, more therapeutic trials of IgAN in children are also needed in the future.

Among the 104 clinical trials, almost over 99% were interventional trials, and only one was observational. The majority of these interventional trials were designed with randomized allocation and parallel assignment of intervention model, but the design methods of single-blind or multi-blind were used in only 34% of trials. Concededly, randomized controlled trials (RCTs) play an extremely important role in designing an excellent clinical study and obtaining convincing experimental findings. In addition, experimental designs with inadequate randomization and no masking could cause bias in the intervention effects and expected results of clinical trials [20,21]. Therefore, our efforts are still needed to improve the quality of clinical trial design in the future.

Our findings showed that only 39 trials were in completed status, and seven trials had been terminated. By analyzing the reasons for terminating trials, we found that four trials were terminated due to difficulties for enrollment, one due to business reasons, one because of lack of effect, and one did not provide the reason. Not only that, only 9% of trials had results, including three trials in terminated status. The ICMJE provided a policy in 2017 that data sharing statements for clinical trials should be advocated for the reason that patients were at risk when participating in clinical trials [22]. They even regarded whether clinical trial reports with results contained data-sharing statements as a condition for considering publication. Obviously, the rate of IPD sharing statement of therapeutic clinical trials for IgAN was low observed in our study. Perhaps our awareness of the importance of data-sharing statements for clinical trials still needs to be raised.

In an analysis of clinical trials with results, only six trials with results were completed. And several common traits were shared in these trials, such as low enrollment and short follow-up time. Notably, these trials did provide new ideas for treating IgAN, such as the effect of Bortezomib on improving renal function and urinary protein of IgAN patients [14], and the application of ACTH Gel in refractory IgAN [15]. Nevertheless, both trials had small sample sizes and no control, thus the exact efficacy needs further validation.

When it comes to the therapeutic strategy for IgAN, supportive therapy remains the foundation of clinical treatment. Among all the supportive managements in our study, the most studied was RAS blockers. Based on the repeated confirmation of many clinical studies, the positively protective role of ACEI and ARB in treating patients with IgAN has been well-recognized, which can effectively control blood pressure and decrease urinary protein levels [3,23]. Nevertheless, in patients with persistent high proteinuria levels or deteriorating renal function, supportive treatment alone may not effectively improve the condition and suppress disease progression. Moreover, the Kidney Disease Improving Global Outcomes (KDIGO) Clinical Practice Guideline for Glomerulonephritis proposed that IgAN patients at risk of progressing to CKD might be considered to receive a six-month course of steroid therapy in addition to maximum supportive treatment [24]. Immunosuppression was the most investigated therapeutic strategy in these trials included in our study, especially steroid therapy. Immune-mediated damage is mainly accountable for the pathogenesis of IgAN. However, currently, the role of steroids in the therapy of IgAN remains a little controversial, which might primarily result from the inevitable adverse events related to medication. Encouragingly, targeted-release budesonide as a novel form of steroid therapy has been a research focus. Nefecon is an oral targeted release steroid, which mainly acts on the terminal ileum to exert immunosuppressive effects, and thereby the AEs of systemic steroid therapy are alleviated to some extent [2,25].

At present, three pathways of complement activation, namely the classical (CP), alternative (AP), and lectin (LP) pathways, have been well-understood. Recently, a variety of novel targeted-therapy drugs for IgAN have been increasingly getting attention from researchers. Complement activation plays an important role in the pathogenesis of IgAN, especially the AP and LP. Generally exploring the four-hit hypothesis of IgAN pathogenesis, increasing the level of serum galactose deficient IgA1 (gd-IgA1) is the first hit. Gd-IgA1 can combine with some autoantibodies causing the production of circulating immune complexes (ICs), which may deposit in the mesangial region of the kidney and cause the activation of mesangial cells, further engendering complement activation, inflammation and immune responses, and a series of immune-related renal damage [17,26]. Complement activation may enhance the damage effects of deposited IgA1 mentioned above on the kidney. Additionally, complement activation in circulation or kidney may increase the intrarenal inflammation responses, resulting in the aggravation of kidney damage and loss of kidney function [5]. Factor B (FB) is an essential component of complement activation by AP. LNP023 is an agent that can selectively block FB, thus preventing the complement activation by AP, which may contribute to alleviating disease progression [25,27]. OMS721 is an antibody targeting MASP-2, a protease involved in the complement activation by LP. It can selectively combine with MASP-2, which may block the role of MASP-2 in activating complement [28]. In addition, a prior study also suggested that the LP of complement activation was associated with pathogenesis and more serious renal injury in IgAN [29]. Whereas, the targeted therapeutic strategy of complement in IgAN is not yet well-established, currently, thus more RCTs are needed to explore its clinical availability.

In our study, the level of proteinuria and change from baseline of proteinuria is the most major inclusion criteria and studied outcome measure, respectively. Many prior findings indicated that high blood pressure and the existence of proteinuria (especially the level >1 g/24 h) were the main conditions that led to a poor prognosis of IgAN [30,31]. Thus, blood pressure control and urine protein decrease are the general goals of therapeutic strategies of IgAN. eGFR was also comparatively main included filtering criteria in these trials, which might be based on previous findings that IgAN patients with decreased baseline eGFR were at higher risk of progression to ESRD or death in the future [32], and different therapeutic strategies might be formulated in patients with diverse renal function. Nonetheless, some problems confused us a little during our analysis of inclusion criteria and outcome measures of these trials as follows: Urine samples in some trials have not specified the methods of collection (random urine or 24-h urine); remission rate (CR/PR), as a main short-term outcome measure in clinical, was not defined in detail in many trials, and the definition of CR varies among trials that explained the definitions. Therefore, improvement should be put in the quality of registration information for clinical trials.

In conclusion, characteristics of therapeutic clinical trials for IgAN registered on ClinicalTrials.gov were comprehensively analyzed and investigated in this study. The number of registered trials had gradually increased over the past decade. Most of these trials were interventional, mainly included adults, and had various observation periods. However, only a few trials were completed status, even fewer trials had results. In the perspective of experiment design, most interventional trials employed randomized allocation and parallel interventional model, but masking was used in less than half of the trials, and IPD sharing statements were also rarely contained in these trials. RAS blockade and immunosuppression were the primarily studied therapeutic strategies. Novel targeted therapy agents were increasingly becoming the research focus. Through our comprehensive analysis of these trials, the current situation of the therapeutic clinical trials for IgAN was generally understood. We expect that more large-scale, multicenter RCTs are designed and carried out to improve the treatment strategy of IgAN in the future. In addition, IPD sharing statements and uploading the latest results should be actively advocated.

Undeniably, some limitations exist in our study. First of all, all the analyzed clinical trials in this study are only from the ClinicalTrials.gov database, which may lead to the deficiency of trials registered with other register platform databases. Secondly, a complete and precise analysis may not be provided because of the lack of registered information in some trials. Thirdly, the effectiveness of the therapeutic clinical trials for IgAN was not compared in this study due to the not excellent design, low enrollment, or short observational period in some trials. Finally, our study cannot always contain in real-time due to the database of clinical trials from ClinicalTrials.gov keeps being constantly updated.

## 5. Conclusion

To sum up, this study mainly investigated the characteristics and current situation of IgAN-related therapeutic clinical trials registered on ClinicalTrials.gov. Our results suggested that adults were the main subjects of these trials, and enrollment was generally relatively low. Interventional trials accounted for the majority of study types, characterized by predominant randomized allocation, parallel intervention model, but insufficient blinding. The rate of being completed, having results and IPD sharing statement is pretty low in these trials. Thus, therapeutic trials for children with IgAN maybe still need in the future. Additionally, more focus should be put on improving the quality of clinical trials, such as designing more large-scale and multicenter RCTs, increasing the publication of experimental results and IPD sharing statements, and so on.

## Data Availability

The data used to support the results of our study are available from the corresponding author upon request.
